# Turbofan Engine Remaining Useful Life Prediction with Reliable Prediction Intervals via LSTM-Based Quantile Regression and Conformal Calibration

**DOI:** 10.3390/s26072249

**Published:** 2026-04-05

**Authors:** Runsheng Diao, Mingzhe Zhou, Guanglei Meng, Shanze Wang

**Affiliations:** School of Automation, Shenyang Aerospace University, Shenyang 110136, China; diaorunsheng@stu.sau.edu.cn (R.D.); wangshanze@stu.sau.edu.cn (S.W.)

**Keywords:** LSTM, quantile regression, uncertainty quantification, CQR, remaining useful life prediction

## Abstract

To overcome the inability of point estimates to adequately characterize uncertainty and the unstable coverage of prediction intervals in turbofan engine remaining useful life (RUL) prediction, this study proposes an LSTM-based quantile regression framework (LSTM-QR). The framework generates a point prediction together with upper and lower predictive bounds in a single forward pass, thereby directly constructing a prediction interval with a nominal coverage of 80%. During training, a weighted pinball loss and an overestimation penalty are introduced to improve the robustness of quantile estimation. During inference, Conformalized Quantile Regression (CQR) is further applied for post hoc interval calibration. Experiments on the NASA C-MAPSS dataset show that the proposed method maintains stable point-prediction performance while substantially improving interval reliability after calibration. Under the same operating condition, PICP increases from 0.590 ± 0.035 to 0.800 ± 0.026 for FD001 → FD001 and from 0.722 ± 0.050 to 0.793 ± 0.032 for FD002 → FD002, corresponding to gains of 21.0 and 7.1 percentage points, respectively, with calibrated RMSE values of 16.235 ± 1.297 and 18.323 ± 0.411. Under cross-condition transfer, where the raw intervals exhibit clear under-coverage, CQR further raises PICP from 0.696 ± 0.046 to 0.806 ± 0.032 for FD001 → FD002 and from 0.593 ± 0.071 to 0.803 ± 0.021 for FD002 → FD001, corresponding to gains of 11.0 and 21.0 percentage points, respectively, while preserving RMSE at 21.758 ± 1.208 and 17.562 ± 0.062. These results indicate that the proposed method provides more reliable and interpretable prediction intervals under varying operating conditions, thereby offering effective support for predictive maintenance decision-making.

## 1. Introduction

With the widespread use of turbofan engines in aviation, marine, and industrial systems, unplanned downtime caused by equipment failure can substantially increase maintenance costs and reduce system availability. Predictive maintenance (PdM) identifies signs of degradation before failure through continuous condition monitoring and trend analysis, thereby enabling maintenance to be scheduled proactively. Compared with corrective or fixed-interval maintenance, PdM can reduce the risk of unexpected failures while avoiding unnecessary maintenance costs. Within the PdM framework, remaining useful life (RUL) prediction is a key task that aims to estimate the time remaining before a system reaches a failure threshold [1]. In terms of modeling paradigms, RUL prediction methods can generally be divided into two categories: physics-based degradation models and statistical or machine-learning models based on historical monitoring data. For engine systems with complex structures and degradation mechanisms that are difficult to model, data-driven methods require less prior knowledge and can learn degradation patterns directly from multisource sensor data. As a result, they have become an important research direction in recent years [2].

Data-driven RUL prediction typically involves data acquisition, health indicator (HI) construction, and life prediction modeling. Traditional approaches often rely on handcrafted features or manually designed HIs, followed by regression using models such as support vector machines or random forests. In contrast, deep learning can automatically learn degradation representations from multivariate time series, thereby reducing the uncertainty and effort associated with feature engineering. In recent years, recurrent neural networks (RNNs) and their variants have been widely applied to RUL prediction. Among these models, long short-term memory (LSTM) networks introduce explicit memory cells through gating mechanisms, effectively alleviating the vanishing-gradient problem encountered by conventional RNNs when learning long-term dependencies. As a result, LSTM can capture latent states that reflect degradation evolution and has shown promising performance in various engine life prediction tasks [3,4].

The existing literature indicates that condition-monitoring-driven prognostics and health management (PHM) has developed into a relatively systematic methodological framework. Relevant review studies have summarized the major components and challenges of PdM and condition-based maintenance (CBM), while also categorizing engine prognostics, degradation modeling, and data-driven methods [5,6,7]. Regarding public benchmarks, NASA developed turbofan engine degradation datasets using the C-MAPSS simulation tool, and the PHM 2008 data challenge further promoted standardized and reproducible algorithm evaluation [8,9,10]. Early studies attempted to learn direct mappings from multisensor sequences to RUL using models such as RNNs [11,12,13,14]. With advances in end-to-end representation learning, numerous deep models have been developed for feature extraction and temporal modeling, including deep CNN-based degradation representation learning [15,16], LSTM-based life regression, and more recent architectures incorporating attention mechanisms and Transformer-based structures [17]. In addition, to address distribution shift caused by changing operating conditions, researchers have explored transfer learning and domain adaptation strategies to improve generalization in cross-condition scenarios [18,19,20,21,22,23].

Despite these advances, most existing RUL studies still emphasize point prediction accuracy, while paying relatively limited attention to interval reliability and deployment robustness under operating-condition shift. In particular, prediction intervals derived from quantile regression may suffer from coverage mismatch under finite-sample conditions and domain shift, which limits their practical value for maintenance-oriented decision-making. Therefore, a reliability-oriented interval prognostics framework is needed to provide both informative uncertainty estimates and improved robustness across operating conditions.

Recent reliability-oriented PHM studies have increasingly emphasized that useful prognostics should not stop at point prediction, but should provide uncertainty-aware outputs that can be translated into maintenance decisions. For example, a recent study in Reliability Engineering & System Safety developed a multi-action predictive maintenance framework for lithium-ion batteries by coupling probabilistic RUL prediction with downstream maintenance optimization, thereby explicitly linking RUL uncertainty to maintenance actions for single- and multi-component systems [24]. In parallel, recent intelligent diagnosis studies have also moved toward reliability-aware learning under practical uncertainty and distribution shift. Representative examples include a multi-source sensor correlation adaptive fusion framework with uncertainty quantification for intelligent fault diagnosis, an evidential deep-learning-based test-time adaptation method for online machinery fault diagnosis, and a multi-source domain adaptation network based on transferable domain attributes and features for cross-domain fault diagnosis [25,26,27]. These studies collectively indicate that reliability, uncertainty awareness, and robustness to domain shift are becoming central concerns in PHM-related data-driven modeling.

However, existing studies differ in task scope and application focus. The above battery-oriented work emphasizes downstream maintenance optimization based on probabilistic RUL outputs, whereas the present study focuses on the upstream prognostic module for turbofan engines. More specifically, this study is concerned with how to construct statistically meaningful and practically deployable RUL prediction intervals under operating-condition shift, so as to provide more trustworthy uncertainty-aware inputs for downstream maintenance decision-making. In this sense, the present work is positioned as a reliability-oriented interval prognostics approach for aero-engines rather than a complete prescriptive maintenance optimization framework.

Motivated by this gap, this study proposes a reliability-oriented LSTM-based quantile regression framework with post hoc conformal calibration for turbofan engine RUL prediction. The framework jointly outputs a point estimate and the lower and upper bounds of a nominal prediction interval. To better reflect maintenance-oriented risk characteristics, low-RUL weighting and a mild overestimation penalty are incorporated during training. In addition, limited target-domain fine-tuning and inference-time conformalized quantile regression (CQR) calibration are introduced to improve interval reliability under operating-condition shift. In this way, the proposed method aims to provide more trustworthy uncertainty-aware prognostic outputs under both same-condition and cross-condition scenarios.

The main contributions of this study are summarized as follows.

(1) A reliability-oriented LSTM-based quantile regression framework is proposed for turbofan engine RUL prediction, which jointly outputs the point estimate and the lower and upper bounds of a nominal prediction interval in a single forward pass. Compared with conventional point-regression-based prognostics, the proposed framework provides more informative uncertainty cues for maintenance-oriented decision-making.

(2) A risk-aware training objective is introduced by combining low-RUL weighting with a mild overestimation penalty. The former increases the optimization emphasis on maintenance-critical near-failure samples, while the latter explicitly suppresses overly optimistic life prediction from a safety perspective.

(3) To improve deployment reliability under operating-condition shift, the framework combines limited target-domain fine-tuning with inference-time CQR calibration. This design enables the model to retain useful point prediction capability while bringing the empirical interval coverage closer to the nominal target under cross-condition scenarios.

(4) Extensive experiments on the NASA C-MAPSS dataset, including same-condition evaluation, cross-condition transfer, ablation analysis, and multi-seed validation, demonstrate that the proposed framework achieves a favorable balance among point prediction accuracy, interval reliability, and practical interpretability.

The remainder of this paper is organized as follows. Section 2 introduces the theoretical foundations of LSTM and quantile regression. Section 3 presents the structure, loss function, and training procedure of LSTM-QR. Section 4 describes the experimental settings and reports the results under both same-condition and cross-condition scenarios. Section 5 discusses the limitations of cross-condition prediction and possible directions for improvement. Section 6 concludes the paper and outlines future work.

## 2. Materials and Methods

### 2.1. LSTM Fundamentals

RNNs are prone to gradient vanishing and explosion when trained on long sequences, which makes learning long-term dependencies difficult. LSTM introduces explicit memory cells through gating mechanisms to alleviate this problem and is therefore well suited for degradation modeling and RUL prediction tasks [28,29].

Given the input $x_{t}$, the previous hidden state $h_{t-1}$, and the previous cell state $c_{t-1}$, a standard LSTM cell is updated as follows:

(1) $$f_{t}=\sigma (W_{f}[h_{t-1},x_{t}]+b_{f}),$$

(2) $$i_{t}=\sigma (W_{i}\cdot [h_{t-1},x_{t}]+b_{i}),$$

(3) $${\tilde{c}}_{t}=\tanh(W_{c}[h_{t-1},x_{t}]+b_{c}),$$

(4) $$c_{t}=f_{t}⊙c_{t-1}+i_{t}⊙{\tilde{c}}_{t},$$

(5) $$o_{t}=\sigma (W_{o}[h_{t-1},x_{t}]+b_{o}),$$

(6) $$h_{t}=o_{t}⊙\tanh(c_{t}).$$

Here, σ(⋅) denotes the sigmoid function, tanh(⋅) denotes the hyperbolic tangent function, ⊙ represents the Hadamard product, and $\left[h_{t-1},x_{t}\right]$ denotes vector concatenation. Through the update mechanism defined in Equations (2)–(5), LSTM uses the cell state to preserve long-term memory and selectively regulates information flow through the forget, input, and output gates. By retaining and updating historical information in a gated manner, LSTM alleviates the vanishing-gradient problem commonly encountered by standard RNNs when modeling long sequences. This architecture can therefore learn discriminative long-term dependency features from degradation sequences and is well suited for RUL prediction.

**Figure 1:**
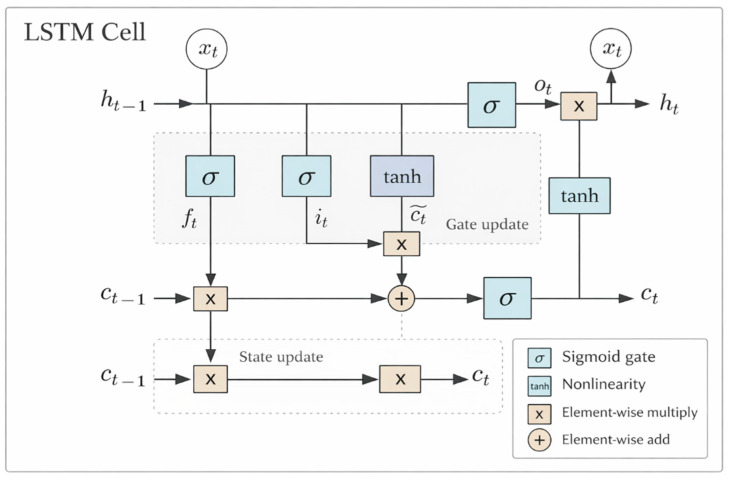
Schematic of a basic LSTM cell.

Figure 1 illustrates the architecture of an LSTM cell. The forget gate controls the retention of historical information, the input gate regulates the incorporation of current information, and the output gate determines how the cell state contributes to the hidden state. Through these gating mechanisms, LSTM can model long-term dependencies in degradation processes more effectively and stably [30].

### 2.2. Quantile Regression and Prediction Intervals

Quantile regression is a widely used uncertainty quantification method for regression tasks. Rather than estimating only the conditional mean, it estimates the conditional quantiles of the response variable given the input. Let Y denote the remaining useful life, and let X denote the input feature vector. The conditional quantile of Y given X=x at quantile level τ∈(0,1) is defined as follows [31]:

(7) $$Q_{\tau }(Y|X=x)=\inf\{y:F_{Y|X}(y|x)\geq \tau \}$$

where $F_{Y|X}(y|x)$ denotes the conditional distribution function. According to Equation (7), when the input is x, $q_{\tau }(x)$ satisfies $P(Y\leq q_{\tau }(x)|X=x)=\tau$, meaning that the probability mass to the left of this point equals τ.

In practice, quantile regression usually learns quantile estimates by minimizing the pinball loss. Given a sample $\left(x_{i},y_{i}\right)$ and model output ${\hat{q}}_{\tau }(x_{i})$, let the error be $e_{i}=y_{i}-{\hat{q}}_{\tau }(x_{i})$.

The pinball loss is written as [31]:

(8) $$L_{\tau }(y_{i},{\hat{q}}_{\tau }(x_{i}))=\max\left(\tau e_{i},(\tau -1)e_{i}\right)=\left\{\begin{array}{ll} \tau (y_{i}-{\hat{q}}_{\tau }(x_{i})), & y_{i}\geq {\hat{q}}_{\tau }(x_{i})\\ (1-\tau )({\hat{q}}_{\tau }(x_{i})-y_{i}), & y_{i}<{\hat{q}}_{\tau }(x_{i}) \end{array}\right.$$

This loss imposes asymmetric penalties on underestimation and overestimation, such that its optimum statistically corresponds to the target quantile. It can therefore be used to learn prediction bounds under different risk preferences.

To obtain both point estimates and interval information simultaneously, this study adopts joint learning of multiple quantiles. Let τ∈{0.1,0.5,0.9}. In one forward pass, the model outputs ${\hat{q}}_{0.1}(x)$, ${\hat{q}}_{0.5}(x)$ and ${\hat{q}}_{0.9}(x)$. Here, ${\hat{q}}_{0.5}(x)$, i.e., the median, is commonly used as a robust point estimate, while $\left[{\hat{q}}_{0.1}(x),{\hat{q}}_{0.9}(x)\right]$ forms a central 80% prediction interval (PI). It should be emphasized that the statistical meaning of an 80% interval is that, under ideal conditions, it covers future samples with probability approximately 1−α=0.8 [32,33].

(9) $$P\left(Y\in [{\hat{q}}_{\alpha /2}(X),{\hat{q}}_{1-\alpha /2}(X)]\right)\approx 1-\alpha ,\;\alpha =0.2$$

This interpretation provides a range for maintenance decision-making, spanning from a conservative lower bound to an optimistic upper bound: the former reflects the risk of earlier failure, whereas the latter reflects the possibility of later failure.

Two issues merit attention. First, quantile crossing may occur because different quantiles are optimized independently under their respective loss terms. Under finite-sample conditions, occasional violations such as ${\hat{Q}}_{0.1}(x)>{\hat{Q}}_{0.9}(x)$ can lead to invalid interval definitions. In practice, this issue can be addressed by post hoc monotonic reordering or by introducing monotonicity constraints during training. Second, coverage bias may still arise even when the quantiles are estimated correctly. Under finite-sample conditions or distribution shift, nominal coverage may deviate from empirical coverage, resulting in under-coverage or excessively conservative intervals. Therefore, a distribution-free post hoc calibration method is further employed in this study to align empirical coverage more closely with the target level and thereby improve interval reliability in cross-condition scenarios [34].

### 2.3. Evaluation Metrics

To quantitatively evaluate prediction interval quality, two commonly used metrics are interval coverage and interval width. The prediction interval coverage probability (PICP) denotes the proportion of ground-truth values that fall within the predicted intervals and reflects interval reliability. The mean prediction interval width (MPIW) denotes the average width of the predicted intervals and reflects interval sharpness. Suppose that there are N test samples. For the i-th sample, let the predicted lower and upper bounds be $L_{i}$ and $U_{i}$, respectively, and let $y_{i}$ denote the ground-truth value [35]:

Coverage metric:

(10) $$PICP=\frac{1}{N}\sum_{i=1}^{N}I\left(y_{i}\in [{\hat{L}}_{i},{\hat{U}}_{i}]\right)$$

Mean interval width:

(11) $$MPIW=\frac{1}{N}\sum_{i=1}^{N}\left({\hat{U}}_{i}-{\hat{L}}_{i}\right)$$

where ${\hat{L}}_{i}={\hat{q}}_{0.1}(x_{i})$ and ${\hat{U}}_{i}={\hat{q}}_{0.9}(x_{i})$.

For a given nominal confidence level (e.g., 80%), the ideal PICP should be close to 0.8. Provided that coverage is adequate, a smaller MPIW indicates a tighter prediction interval. Interval quality can also be evaluated using composite metrics that penalize insufficient coverage. In the following experiments, PICP and MPIW are reported to compare the uncertainty quantification performance of different models. When PICP is markedly lower than the nominal level, the interval exhibits under-coverage; when it is substantially higher, the interval becomes overly conservative [36].

### 2.4. Point Prediction Metrics

In addition to uncertainty metrics, point prediction accuracy remains a fundamental requirement for life prediction models. In this study, the root mean square error (RMSE) is used to quantify the average prediction error on the test set and is defined as follows:

(12) $$RMSE=\sqrt{\frac{1}{N}\sum_{i=1}^{N}({\hat{y}}_{i}-y_{i}{)}^{2}}$$

where ${\hat{y}}_{i}$ denotes the predicted RUL of the i-th sample and $y_{i}$ denotes the true RUL. RMSE treats early and late prediction errors symmetrically. In real maintenance decision-making, however, the consequences of these two types of errors are asymmetric: late predictions are generally more hazardous because they may delay maintenance and increase the risk of failure. To reflect this asymmetry, the PHM 2008 challenge introduced an asymmetric scoring function, commonly referred to as the PHM Score, which imposes a heavier penalty on late predictions. The sample-wise PHM Score is defined as follows:

(13) $$d_{i}={\hat{y}}_{i}-y_{i},s_{i}=\left\{\begin{array}{ll} \exp\left(-\frac{d_{i}}{13}\right)-1, & d_{i}<0\\ \exp\left(\frac{d_{i}}{10}\right)-1, & d_{i}\geq 0 \end{array}\right.$$

Here, $d_{i}$ denotes the prediction error of the i-th sample. When $d_{i}\geq 0$, the model yields a late prediction and incurs a larger exponential penalty; when $d_{i}<0$, the penalty is relatively smaller. The overall PHM Score can be computed as either the sum or the mean of the sample-wise scores. In this study, the mean form is adopted to facilitate comparisons across test sets of different sizes. A lower PHM Score indicates better performance under a maintenance-oriented risk preference. In the following cross-condition experiments, both RMSE and PHM Score are reported to comprehensively evaluate point prediction performance and the control of late-prediction risk.

## 3. Proposed Framework

### 3.1. Overall Framework and Task Definition

This study addresses the uncertainty quantification requirement in turbofan engine remaining useful life (RUL) prediction by constructing prediction intervals with empirically calibrated coverage while simultaneously providing point estimates. Unlike conventional regression methods that output only a single RUL estimate, the proposed framework jointly considers point prediction accuracy and interval reliability, where the former reflects life estimation error and the latter reflects interval coverage and sharpness. In this way, the model can provide more informative risk cues for maintenance-oriented decision-making.

In terms of task settings, both same-condition and cross-condition scenarios are considered. The same-condition scenario is used to evaluate the baseline performance of the model when the data distribution is relatively consistent, whereas the cross-condition scenario is used to assess generalization ability and interval reliability under distribution shift. Under cross-condition transfer, differences in operating-condition combinations and sensor response distributions may induce point prediction bias and interval under-coverage. Therefore, limited target-domain supervision and post hoc interval calibration are introduced to improve prediction reliability.

Let $\{x_{t}{\}}_{t=1}^{T}$ denote the multivariate time series of a unit, where $x_{t}\in R^{d}$ is the observation vector at cycle t. Sliding windows are used to construct input samples, such that consecutive observations form an input sequence and the corresponding RUL at the end of the window is predicted. The model uses an LSTM encoder to extract temporal degradation features and a quantile regression head to jointly output three quantiles, namely ${\hat{q}}_{0.1}$,${\hat{q}}_{0.5}$ and ${\hat{q}}_{0.9}$. The median quantile ${\hat{q}}_{0.5}$ is used as the point prediction, whereas $\left[{\hat{q}}_{0.1},{\hat{q}}_{0.9}\right]$ forms the raw prediction interval.

**Figure 2:**
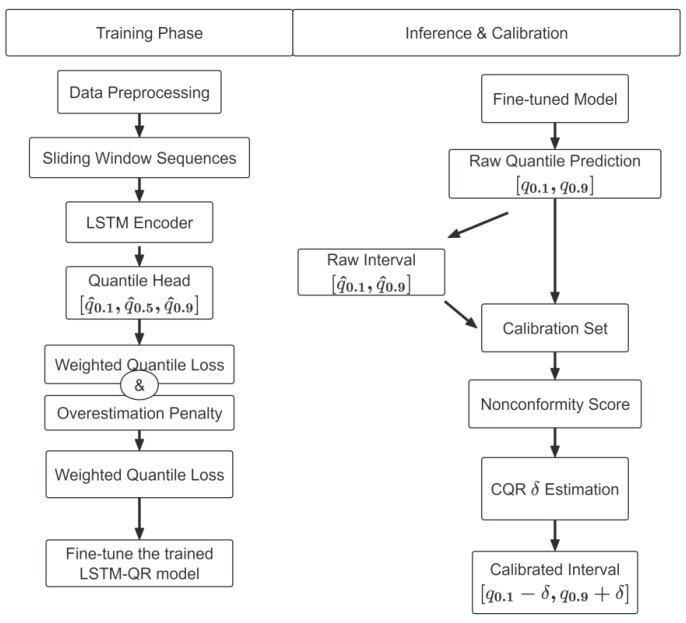
Overall workflow of the proposed method. The pipeline includes source-domain training, target-domain fine-tuning, and inference-time CQR calibration. The model first outputs raw quantile predictions, where the median quantile is used as the point prediction and the lower and upper quantiles form the raw prediction interval. CQR then expands the raw interval based on the calibration set to improve empirical coverage consistency.

Figure 2 illustrates the overall workflow of the proposed method. The pipeline consists of three stages. First, in the training phase, source-domain data are preprocessed and transformed into sliding-window sequences, which are then fed into the LSTM-QR model. The model is optimized using the weighted quantile loss together with an overestimation penalty to improve both quantile estimation quality and maintenance-oriented risk control. Second, under cross-condition settings, the trained model is further fine-tuned on a small labeled target-domain set to alleviate systematic errors caused by domain shift. Third, during inference, the fine-tuned model outputs raw quantile predictions, from which the raw interval is obtained. An independent calibration set is then used to compute nonconformity scores and estimate the CQR correction term δ, so that the raw interval $\left[{\hat{q}}_{0.1},{\hat{q}}_{0.9}\right]$ is expanded into the calibrated interval $\left[{\hat{q}}_{0.1}-\delta ,{\hat{q}}_{0.9}+\delta \right]$. This three-stage design enables the framework to preserve point prediction capability while improving interval reliability under both same-condition and cross-condition scenarios.

### 3.2. LSTM-QR Model Structure

**Figure 3:**
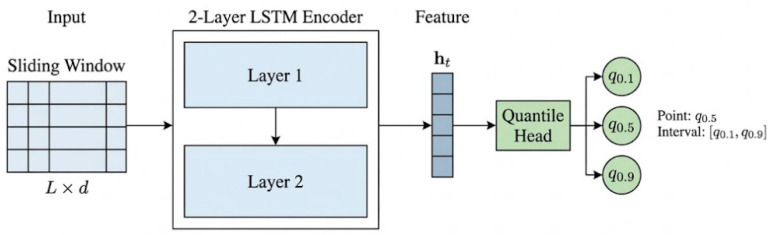
Internal architecture of the proposed LSTM-QR model and illustration of raw prediction interval construction. The LSTM encoder extracts temporal degradation features from sliding-window inputs, and the quantile regression head jointly outputs the lower, median, and upper quantiles.

Figure 3 illustrates the internal architecture of the proposed LSTM-QR model. The model takes multivariate time-series data in the form of sliding windows, encodes them using an LSTM, and jointly outputs multiple quantiles through a quantile regression head, thereby enabling both RUL point prediction and raw prediction interval construction. Let $X_{t}\in R^{L\times d}$ denote the input window, where L is the window length and d is the input feature dimension. Samples within the window are arranged chronologically to characterize the recent degradation dynamics of the equipment. The LSTM encoder models the temporal dependencies within the window, extracts sequential features of degradation evolution, and maps the window to a fixed-dimensional high-level representation. In this study, the hidden state at the final time step is used as the window representation for subsequent quantile regression.

At the output layer, the model jointly predicts three quantiles corresponding to the lower, median, and upper levels, which are set to 0.1, 0.5, and 0.9 in this study. Accordingly, ${\hat{q}}_{0.5}$ serves as the point prediction, whereas ${\hat{q}}_{0.1}$ and ${\hat{q}}_{0.9}$ define the lower and upper bounds of the nominal 80% prediction interval. This design enables the model to directly output interval estimates with explicit coverage semantics while preserving point prediction capability in a single forward pass.

Because mild quantile crossing may occur under finite-sample training, the lower and upper bounds are consistently reordered during metric calculation and visualization; specifically, the smaller value is treated as the lower bound and the larger value is treated as the upper bound, thereby ensuring a valid and semantically consistent interval. It should be emphasized that this subsection describes only the raw quantile output structure of the model. The post hoc CQR procedure used to improve coverage consistency is introduced separately in Section 3.4.

Overall, the LSTM-QR model combines temporal feature encoding with joint quantile output, allowing it to provide RUL point estimates and raw prediction intervals simultaneously. This design forms the structural basis for the subsequent training objective and the inference-time CQR calibration.

### 3.3. Training Objective Design

To jointly predict the lower, median, and upper quantiles, this study adopts multi-quantile learning based on the pinball loss. For a given quantile level τ∈{0.1,0.5,0.9}, let the prediction error be defined as

(14) $$e_{i}^{\tau }=y_{i}-{\hat{q}}_{\tau }(x_{i})$$

where $y_{i}$ is the ground-truth RUL of sample i, and ${\hat{q}}_{\tau }(x_{i})$ denotes the predicted τ-th conditional quantile. The corresponding pinball loss is written as

(15) $$\rho_{\tau }(e_{i}^{\tau })=\max(\tau e_{i}^{\tau },(\tau -1)e_{i}^{\tau })$$

By summing the loss terms over all target quantiles, the model can learn the lower, median, and upper bounds in a single forward pass. This formulation enables simultaneous point prediction and raw prediction interval construction within a unified regression framework.

However, turbofan degradation samples are usually unevenly distributed over the life cycle. In particular, samples from the long-life stage often dominate the training set, whereas low-RUL samples that are more critical for maintenance decisions are relatively sparse. If all samples are treated equally, the model may focus excessively on the abundant early-stage data and underrepresent the degradation behavior near failure. To alleviate this imbalance, an RUL-dependent weight $w_{i}$ is introduced so that samples with smaller true RUL values receive larger weights. In this way, the contribution of near-failure samples is amplified during optimization, improving the learning emphasis on maintenance-critical operating regions. To preserve the overall loss scale and maintain training stability, the weights are mean-normalized so that their average equals one. The weighted multi-quantile loss is therefore defined as

(16) $$L_{w}=\frac{1}{N}\sum_{i=1}^{N}w_{i}\sum_{\tau \in \{0.1,0.5,0.9\}}\rho_{\tau }(e_{i,\tau }).$$

This weighting strategy does not change the statistical meaning of quantile regression itself; rather, it reallocates optimization emphasis toward low-RUL regions that are more relevant to practical prognostics.

In addition to sample imbalance, RUL prediction also exhibits asymmetric engineering risk. In predictive maintenance, overestimating the remaining life may delay maintenance actions and thus cause more serious consequences than a similarly sized underestimation. To reflect this risk preference, a mild overestimation penalty is imposed on the median prediction ${\hat{q}}_{0.5}$, which serves as the point estimate. Specifically, only the part where the predicted median exceeds the true RUL is penalized:

(17) $$L_{over}=\frac{1}{N}\sum_{i=1}^{N}\max({\hat{q}}_{0.5}(x_{i})-y_{i},0).$$

Unlike the weighted quantile loss, which mainly adjusts the learning emphasis across life stages, this term explicitly discourages hazardous late predictions from the perspective of maintenance risk control. Therefore, the two components play different roles: the former improves quantile learning under life-stage imbalance, whereas the latter introduces a safety-oriented bias against RUL overestimation.

The final training objective is defined as

(18) $$L=L_{w}+\lambda L_{over}$$

where λ>0 is a hyperparameter controlling the trade-off between distributional quantile fitting and overestimation suppression. Overall, this risk-aware objective encourages the model to learn more informative interval bounds while reducing the chance of overly optimistic point predictions, thereby making the resulting outputs better aligned with maintenance-oriented decision requirements.

### 3.4. CQR Calibration Method

Although quantile regression can directly produce a nominal prediction interval, the empirical coverage of the raw interval may deviate from the target level under finite-sample conditions and, more importantly, under cross-condition distribution shift. Recent studies have also attempted to improve conditional coverage in quantile-based uncertainty estimation through more structured quantile-learning strategies [37]. Therefore, this study employs Conformalized Quantile Regression (CQR) as a post hoc calibration step at inference time to improve interval reliability. This procedure does not retrain or modify the model parameters. Instead, it adjusts only the interval boundaries in a data-driven manner based on an independent calibration set.

For a test sample $x_{i}$, let the raw quantile outputs of the trained model be ${\hat{q}}_{0.1}(x_{i})$, ${\hat{q}}_{0.5}(x_{i})$ and ${\hat{q}}_{0.9}(x_{i})$ corresponding to the lower bound, median, and upper bound, respectively. The raw prediction interval is defined as $\left[{\hat{q}}_{0.1}(x_{i}),{\hat{q}}_{0.9}(x_{i})\right]$. Because mild quantile crossing may occasionally occur under finite-sample training, the lower and upper bounds are first reordered when necessary so that the smaller value is treated as the lower bound and the larger value is treated as the upper bound. This step ensures a valid raw interval before calibration.

Given an independent calibration set $\{{\left(x_{i},y_{i}\right)\}}_{i=1}^{n}$, the nonconformity score for each sample is defined as the extent to which the ground-truth value falls outside the raw interval [38,39,40]:

(19) $$s_{i}=\max({\hat{q}}_{0.1}(x_{i})-y_{i},\;y_{i}-{\hat{q}}_{0.9}(x_{i}),\;0)$$

A larger score indicates that the raw interval is less compatible with the observed target. Based on the empirical distribution of $\{s_{i}{\}}_{i=1}^{n}$, a calibration value δ is estimated according to the desired coverage level. For a target coverage level of 1−α, the calibration value is defined as the k-th order statistic of the nonconformity scores, where k=[(n+1)(1−α)]-*th*. This quantity represents the amount of interval expansion needed to bring the empirical coverage closer to the desired level.

The calibrated interval for a new sample x is then constructed as

$$\left[{\hat{q}}_{0.1}(x)-\delta ,{\hat{q}}_{0.9}(x)+\delta \right]$$

It should be emphasized that this post hoc calibration changes only the lower and upper interval boundaries and leaves the median prediction ${\hat{q}}_{0.5}(x)$ unchanged. Consequently, point-prediction metrics such as RMSE and PHM Score remain unchanged before and after calibration, whereas interval-related metrics such as PICP and MPIW vary with the interval adjustment. This distinction is important because the role of CQR in this study is not to improve point prediction accuracy, but to correct coverage mismatch and enhance the reliability of predictive intervals.

In the same-condition setting, the raw quantile interval may already achieve empirical coverage close to the nominal level, so the calibration effect is typically limited. In contrast, under cross-condition transfer, distribution shift often causes under-coverage of the raw interval, making calibration more necessary. In this study, a small labeled target-domain set is used not only for fine-tuning but also for building the calibration set, so that the calibration samples are more consistent with the deployment scenario. As a result, CQR provides a simple and distribution-free mechanism to improve interval reliability under changing operating conditions, although this gain is usually accompanied by some interval widening.

## 4. Experiments and Results

### 4.1. Dataset and Implementation Details

The proposed method is validated using the NASA C-MAPSS dataset generated by the C-MAPSS simulation tool developed at NASA Ames Research Center, Moffett Field, CA, USA. All experiments were implemented in Python (version 3.10.19), and the model was developed using PyTorch (version 2.11.0+cu128). Data processing and visualization were performed using NumPy (version 2.2.6), pandas (version 2.3.3), and Matplotlib (version 3.10.8). FD001 and FD002 are selected for both same-condition and cross-condition experiments. Each cycle includes three operating-condition variables and twenty-one sensor variables, resulting in a total of 24 input features. The data split follows the official file structure: train_FD00x is used for training, test_FD00x for testing, and the true terminal RUL values of the test units are provided in RUL_FD00x. Training labels are generated by backtracking from the failure cycle, whereas test labels for the full trajectories are generated by backtracking from the provided terminal RUL values. To stabilize optimization and mitigate the long-tail effect in the early-life stage, the RUL target is uniformly capped at 125 cycles.

For clarity and engineering interpretability, Table 1 summarizes the common physical abbreviations, descriptions, and units of the 21 sensor variables.

Preprocessing includes z-score normalization and the removal of near-constant features; an additional $1\times 10^{-8}$ is introduced during normalization to avoid division by zero. For same-condition experiments, scaler_mode = source is adopted, meaning that the target domain reuses the source-domain normalization statistics. For cross-condition experiments, scaler_mode = per_domain is used, meaning that the target domain applies its own normalization statistics, while the source-domain feature mask is retained to ensure dimensional consistency and mitigate scale shift. The training procedure begins with source-domain pretraining using a unit-level validation split (val_ratio = 0.1), and the best checkpoint is selected accordingly. When finetune_ratio > 0, supervised fine-tuning is performed on a subset of target-domain training units. Adam is used as the optimizer (weight decay = $1\times 10^{-5}$), together with gradient clipping (max-norm = 1.0).

The model jointly outputs three quantiles and is evaluated using RMSE, the PHM 2008 Score, PICP, and MPIW. When CQR is enabled, a calibration set is constructed from randomly truncated windows sampled from target-domain training trajectories to estimate δ, after which the lower and upper interval bounds are symmetrically expanded. Therefore, CQR does not alter the median quantile prediction; accordingly, RMSE and the PHM 2008 Score remain unchanged, whereas PICP and MPIW vary with interval adjustment. The default hyperparameters are seq_len = 30 and batch_size = 256. A two-stage learning-rate schedule is adopted, with $1\times 10^{-3}$ for pretraining and $1\times 10^{-4}$ for fine-tuning. The best model is selected by monitoring the RMSE of the median quantile on the source-domain validation set. The numbers of training epochs are set to 25/30 (pretraining/fine-tuning) for same-condition experiments, 60/50 for FD001 → FD002, and 70/70 for FD002 → FD001 to ensure adequate convergence.

### 4.2. Same-Condition Results and Analysis

Table 2 summarizes the results under the same operating condition. To reduce the influence of stochastic factors such as random initialization and data shuffling, all values are reported as the mean ± standard deviation over three random seeds. Overall, the proposed method achieves stable performance in both same-condition settings, and FD001 remains easier than FD002 in terms of both point prediction and interval estimation. Specifically, FD001 → FD001 achieves RMSE = 16.235 ± 1.297, PHM Score = 394.9 ± 5.0, PICP = 0.800 ± 0.026, and MPIW = 33.766 ± 0.811, whereas FD002 → FD002 achieves RMSE = 18.323 ± 0.411, PHM Score = 1658.1 ± 367.2, PICP = 0.793 ± 0.032, and MPIW = 37.203 ± 3.583. These results indicate that the model can learn a more stable degradation mapping under a single operating condition, while the more complex multi-condition setting of FD002 leads to higher prediction error and greater interval uncertainty.

**Figure 4:**
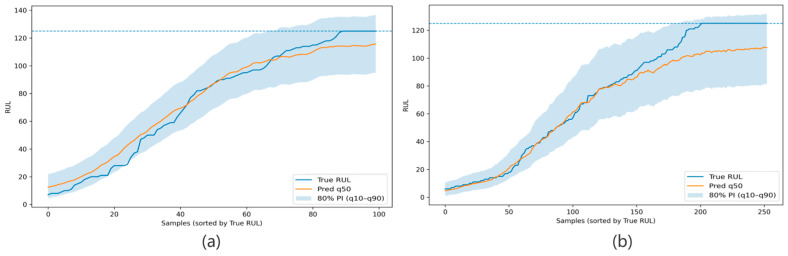
Sorted ribbon plots of test results under the same operating condition. Panels (**a**,**b**) correspond to the results for FD001 and FD002, respectively. The blue dashed line indicates the capped maximum RUL value of 125 cycles used in the data preprocessing.

**Figure 5:**
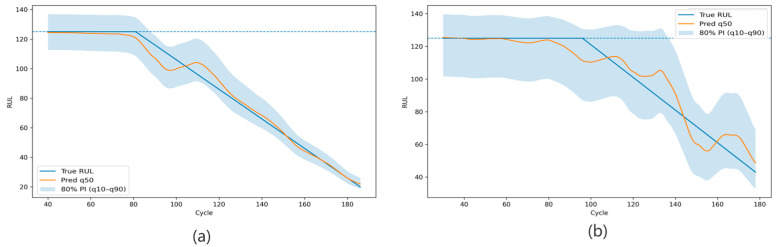
RUL degradation trajectories of representative engines under the same operating condition. Panels (**a**,**b**) show the representative engine trajectories for FD001 and FD002, respectively. The blue dashed line indicates the capped maximum RUL value of 125 cycles used in the data preprocessing.

**Figure 6:**
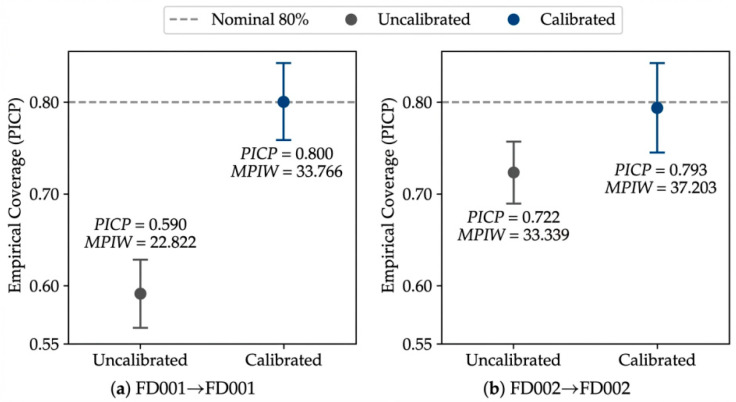
Interval calibration diagnostics at the nominal 80% level under the same-condition setting. Error bars denote 95% confidence intervals of the empirical coverage. The dashed horizontal line indicates the nominal coverage level of 0.80. Panels (**a**,**b**) correspond to FD001 → FD001 and FD002 → FD002, respectively.

**Figure 7:**
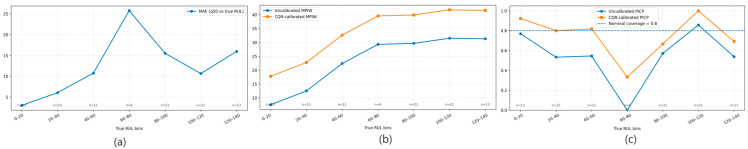
Prediction error and interval behavior across different life stages on FD001. Panels (**a**–**c**) show the binned mean absolute error (MAE), mean prediction interval width (MPIW), and prediction interval coverage probability (PICP), respectively, as functions of the true RUL.

**Figure 8:**
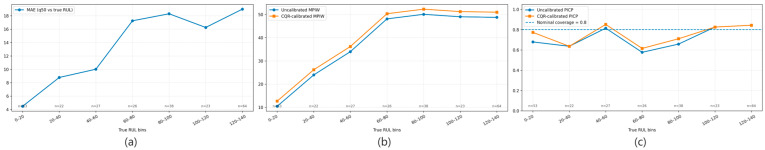
Prediction error and interval behavior across different life stages on FD002. Panels (**a**–**c**) show the binned mean absolute error (MAE), mean prediction interval width (MPIW), and prediction interval coverage probability (PICP), respectively, as functions of the true RUL.

From the perspective of statistical stability, the standard deviations under the same-condition setting are generally moderate, suggesting that the proposed framework is not overly sensitive to random seeds when the training and test distributions are matched. In particular, the PICP values of both FD001 and FD002 remain close to the nominal 80% level across repeated runs, indicating that the calibrated intervals maintain reliable coverage with limited fluctuation. Although FD002 exhibits a larger variation in PHM Score and MPIW than FD001, this behavior is consistent with its higher complexity and stronger operating-condition heterogeneity. Therefore, the multi-seed results further support the conclusion that the proposed method provides stable point prediction performance and reasonably reliable prediction intervals under same-condition scenarios. Figure 4, Figure 5, Figure 6, Figure 7 and Figure 8 further visualize the prediction behavior under the same-condition setting. Specifically, Figure 4 presents the sorted ribbon plots, Figure 5 shows representative engine degradation trajectories, Figure 6 reports the interval calibration diagnostics, and Figure 7 and Figure 8 summarize the stage-wise prediction error and interval behavior for FD001 and FD002, respectively.

### 4.3. Cross-Condition Results and Analysis

To evaluate generalization under changing operating-condition distributions, cross-condition experiments are conducted in both directions, FD001 → FD002 and FD002 → FD001, and the results are summarized in Table 3. Similarly to Table 2, all values are reported as the mean ± standard deviation over three random seeds. Under distribution shift, the proposed framework achieves RMSE = 21.758 ± 1.208, PHM Score = 2943.7 ± 782.6, PICP = 0.806 ± 0.032, and MPIW = 50.952 ± 0.462 for FD001 → FD002, while for FD002 → FD001 it achieves RMSE = 17.562 ± 0.062, PHM Score = 633.3 ± 12.7, PICP = 0.803 ± 0.021, and MPIW = 40.888 ± 2.336. Compared with the same-condition setting, the cross-condition results show larger performance variability, which is expected because operating-condition shift affects both point prediction bias and interval calibration difficulty.

**Figure 9:**
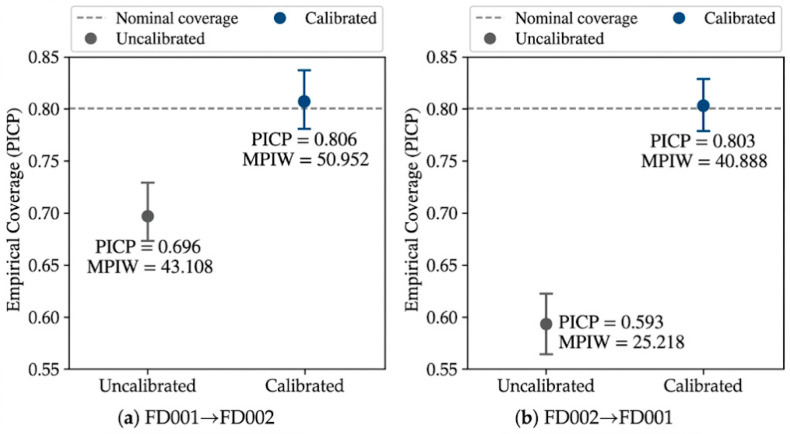
Interval calibration diagnostics at the nominal 80% level under the cross-condition setting.

**Figure 10:**
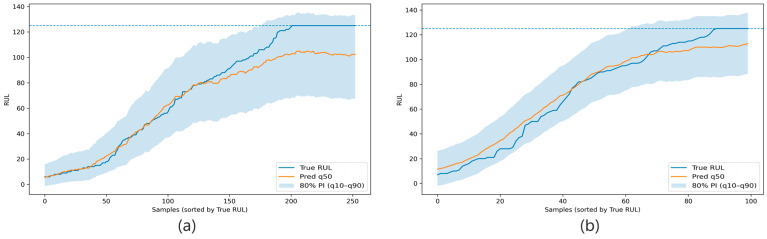
Sorted ribbon plots of test results under the cross-condition setting. Panels (**a**,**b**) correspond to the results for FD001 → FD002 and FD002 → FD001, respectively. The blue dashed line indicates the capped maximum RUL value of 125 cycles used in the data preprocessing.

**Figure 11:**
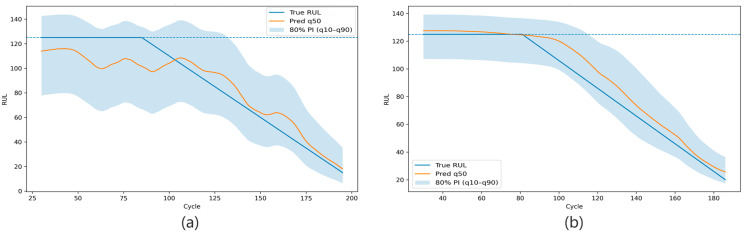
RUL degradation trajectories of representative engines in the cross-condition setting. Panel (**a**) shows the prediction trajectory of a representative engine under FD002 → FD001, and panel (**b**) shows the prediction trajectory of a representative engine under FD001 → FD002. The blue dashed line indicates the capped maximum RUL value of 125 cycles used in the data preprocessing.

Nevertheless, an important observation is that the empirical coverage remains consistently close to the nominal 80% level in both transfer directions. This suggests that the combination of limited target-domain fine-tuning and inference-time CQR calibration is effective in improving interval reliability even when the source and target distributions are mismatched. At the same time, the two transfer directions still exhibit noticeable asymmetry. FD001 → FD002 shows a larger PHM Score and a wider interval, whereas FD002 → FD001 achieves a lower RMSE and a narrower interval on average. This indicates that cross-condition transfer difficulty is direction-dependent and that good point prediction performance alone does not necessarily imply well-calibrated uncertainty estimation. Overall, the multi-seed results confirm that the proposed framework maintains not only acceptable predictive accuracy but also relatively robust calibrated coverage under cross-condition scenarios. Figure 9, Figure 10 and Figure 11 further visualize the calibration effect and prediction behavior under cross-condition transfer. Figure 9 presents the interval calibration diagnostics, Figure 10 shows the sorted ribbon plots for the two transfer directions, and Figure 11 illustrates representative engine degradation trajectories.

### 4.4. Comparison with Baseline Methods

To further verify the relative advantages of the proposed method in both point prediction accuracy and uncertainty quantification, this subsection compares four representative deep-learning baselines for predictive uncertainty estimation: MC Dropout-LSTM, Gaussian NLL-LSTM, TCN-QR and Deep Ensemble Gaussian-LSTM [41,42,43,44]. All methods are evaluated under the same preprocessing scheme, window construction strategy, and evaluation protocol. RMSE, the PHM 2008 Score, PICP and MPIW for the nominal 80% prediction interval are reported to jointly assess point prediction accuracy, coverage reliability, and interval sharpness. To improve the statistical reliability of the comparison, all competing baselines were also repeated over three random seeds, and the results are reported as the mean ± standard deviation.

The same-condition results are presented in Table 4 and Table 5. On FD001, MC Dropout-LSTM and TCN-QR achieve slightly lower RMSE values than the proposed method, but both suffer from evident under-coverage, with PICP = 0.640 ± 0.020 and 0.700 ± 0.115, respectively. By contrast, Deep Ensemble Gaussian-LSTM achieves higher coverage (PICP = 0.880 ± 0.070), but at the cost of a substantially wider interval (MPIW = 48.612 ± 9.386). Gaussian NLL-LSTM achieves PICP close to the nominal level, but its interval width and variance are both relatively large. Compared with these methods, the proposed LSTM-QR achieves PICP = 0.800 ± 0.026 and MPIW = 33.766 ± 0.811, yielding the most balanced trade-off between coverage reliability and interval sharpness while maintaining competitive point prediction performance.

In the more complex FD002 scenario (Table 5), several baseline methods achieve lower RMSE values than the proposed method, but their interval reliability is either insufficient or less balanced. For example, Gaussian NLL-LSTM and Deep Ensemble Gaussian-LSTM achieve lower RMSE values, but their PICP values remain below the nominal 80% level. In contrast, the proposed LSTM-QR achieves PICP = 0.793 ± 0.032, which is the closest to the target coverage among the compared methods, although its RMSE is not the lowest. These results indicate that under more complex multi-condition settings, the proposed method remains advantageous in terms of interval reliability and overall calibration quality rather than pure point prediction accuracy alone.

The cross-condition results are presented in Table 6 and Table 7. For FD001 → FD002, the proposed LSTM-QR achieves the lowest RMSE among all compared methods (21.758 ± 1.208) while maintaining calibrated coverage close to the nominal level (PICP = 0.806 ± 0.032). Its interval width (MPIW = 50.952 ± 0.462) is also comparable to that of TCN-QR and smaller than those of MC Dropout-LSTM, Gaussian NLL-LSTM, and Deep Ensemble Gaussian-LSTM. These results indicate that, in this transfer direction, the proposed method achieves the best balance between point prediction accuracy and interval reliability, although its PHM Score still leaves room for improvement.

For FD002 → FD001, the advantage of the proposed method becomes even more pronounced. The proposed LSTM-QR achieves RMSE = 17.562 ± 0.062, which is substantially lower than those of all baseline methods, while also maintaining calibrated coverage close to the nominal level (PICP = 0.803 ± 0.021). Moreover, its MPIW = 40.888 ± 2.336 is markedly smaller than those of the competing methods, all of which require much wider intervals to achieve comparable coverage. Although the PHM Score is not the lowest, the proposed method clearly provides the best overall compromise between point prediction accuracy, interval reliability, and interval compactness in this transfer direction.

Overall, the comparative experiments indicate that some baseline methods tend to produce excessively wide intervals in pursuit of higher coverage, whereas others suffer from under-coverage when maintaining relatively narrow intervals. In contrast, the proposed LSTM-QR consistently maintains coverage close to the nominal level and demonstrates stronger overall competitiveness under cross-condition settings, making it more suitable for interval-based RUL prediction in maintenance-oriented applications.

### 4.5. Ablation Study

To clarify the individual roles of low-RUL weighting, the overestimation penalty, fine-tuning, and CQR calibration, ablation experiments were conducted under the two cross-condition settings, namely FD001 → FD002 and FD002 → FD001. The compared variants include Plain QR, Weighting only, Over-penalty only, Full risk-aware, and their corresponding combinations with fine-tuning and CQR. This design allows the separate and joint effects of the four components to be examined under distribution shift.

Three main observations can be drawn from Table 8 and Table 9. First, fine-tuning is the dominant factor for effective cross-condition transfer. Without fine-tuning, all variants perform poorly in both directions. For example, in FD001 → FD002, Plain QR yields RMSE = 51.985 and PICP = 0.261, whereas Plain QR + FT reduces RMSE to 20.725 and improves PICP to 0.806. Similarly, in FD002 → FD001, Plain QR improves from RMSE = 48.645 and PICP = 0.260 to RMSE = 15.297 and PICP = 0.600 after fine-tuning. These results indicate that source-domain quantile learning alone is insufficient under operating-condition shift, and that limited target-domain adaptation is essential for recovering cross-condition predictive capability.

Second, the risk-aware objective mainly reshapes the trade-off between point accuracy and maintenance-oriented risk, rather than uniformly improving all metrics. In FD001 → FD002, Full risk-aware + FT gives a slightly higher RMSE than Plain QR + FT (21.050 vs. 20.725), but achieves a clearly lower PHM Score (2272.3 vs. 3411.8), indicating better control of maintenance-related late-prediction risk. In the same direction, Over-penalty only + FT achieves RMSE = 20.841, PHM Score = 2982.9, and PICP = 0.810, suggesting that the overestimation penalty helps suppress overly optimistic predictions while maintaining competitive point accuracy. In FD002 → FD001, however, weighting only + FT achieves the best RMSE and PHM Score among the non-CQR fine-tuned variants, whereas Over-penalty only + FT attains the highest PICP. These results suggest that low-RUL weighting and overestimation control influence different aspects of prediction behavior, and that their effects are direction-dependent under cross-condition transfer.

Third, CQR mainly serves to correct coverage mismatch, typically at the cost of interval widening. In FD001 → FD002, Plain QR + FT + CQR increases PICP from 0.806 to 0.822, while MPIW increases from 48.055 to 49.831; Full risk-aware + FT + CQR raises PICP from 0.747 to 0.806, with MPIW increasing from 46.830 to 51.438. In FD002 → FD001, the effect is more pronounced: Plain QR + FT + CQR improves PICP from 0.600 to 0.910, but also expands MPIW from 24.994 to 50.090. In comparison, Full risk-aware + FT + CQR achieves PICP = 0.810 with a narrower MPIW of 43.554, suggesting a more balanced compromise between target coverage and interval sharpness. Therefore, CQR should be understood as a post hoc reliability correction step rather than a mechanism for improving point prediction accuracy.

Overall, the ablation study shows that fine-tuning is the key contributor to cross-condition transferability, the risk-aware objective mainly affects the accuracy–risk trade-off, and CQR is primarily responsible for restoring empirical coverage toward the nominal target. Taken together, these components support the proposed framework as a reliability-oriented interval prognostics pipeline rather than a design that uniformly optimizes every metric.

## 5. Discussion

The results suggest that the proposed framework is useful not only for improving predictive accuracy, but also for supporting maintenance-oriented decision-making through interval-aware prognostics. Unlike a single point estimate, the predicted interval provides an explicit risk range for the remaining life. In practice, the lower bound can be interpreted as a conservative estimate associated with earlier failure risk, whereas the upper bound reflects a more optimistic estimate associated with later failure. Therefore, when the lower bound approaches a maintenance threshold, a more cautious inspection or replacement strategy may be preferred; when the interval is wide, the system operator may choose to increase monitoring frequency or combine the prediction with additional diagnostic evidence before making a maintenance decision. In this sense, the proposed method provides more informative support for maintenance planning than point prediction alone.

Another important implication is that reliability calibration is especially necessary under operating-condition shift. The cross-condition results show that raw quantile intervals may suffer from under-coverage, indicating that quantile regression alone cannot guarantee the target confidence level when the deployment distribution differs from the training distribution. In this context, CQR plays a practical role as a lightweight post hoc correction mechanism: it does not change the point prediction itself, but adjusts the interval boundaries so that empirical coverage is brought closer to the nominal level. This property is valuable for deployment because it improves interval reliability without requiring a complete redesign of the prediction model. However, the benefit is typically accompanied by wider intervals, which reflects the inherent trade-off between coverage and sharpness.

It should also be noted that the proposed framework is intended as a reliability-oriented prognostic module rather than a complete maintenance optimization system. In practical industrial settings, interval RUL prediction would need to be further integrated with maintenance thresholds, resource constraints, inspection cost, and mission scheduling. Therefore, the main contribution of this study lies in providing a more reliable uncertainty-aware input for downstream maintenance decision processes, rather than directly solving the full prescriptive maintenance problem.

## 6. Conclusions

To address the limitation of point estimates in turbofan engine remaining useful life prediction, this study proposed an LSTM-based interval prediction framework that jointly outputs point estimates and the bounds of a nominal 80% prediction interval. By combining weighted quantile learning, a mild overestimation penalty, limited target-domain fine-tuning, and post hoc CQR calibration, the proposed method improves interval reliability while preserving point prediction capability.

Experiments on the NASA C-MAPSS dataset showed that the proposed framework achieves a favorable balance between point prediction accuracy and interval quality under both same-condition and cross-condition settings. In particular, the results indicate that fine-tuning is crucial for cross-condition transfer, whereas CQR is effective in correcting coverage mismatch and improving interval reliability under distribution shift.

From a practical perspective, the proposed method can provide more informative support for maintenance-oriented prognostics than point prediction alone by offering a calibrated uncertainty range together with the RUL estimate. Nevertheless, this study is still limited by the use of the C-MAPSS dataset, the absence of explicit monotonicity constraints, and the possible interval widening introduced by calibration. Future work will focus on domain adaptation, conditional calibration, monotonicity-aware learning, and validation on datasets and decision-oriented evaluation protocols that are closer to real engineering practice.

## Figures and Tables

**Table 1:** Description of sensor variables.

Sensor ID	Abbreviation	Description	Unit
s1	T2	Fan inlet total temperature	°R
s2	T24	LPC outlet total temperature	°R
s3	T30	HPC outlet total temperature	°R
s4	T50	LPT outlet total temperature	°R
s5	P2	Fan inlet pressure	psia
s6	P15	Bypass-duct total pressure	psia
s7	P30	HPC outlet total pressure	psia
s8	Nf	Fan physical speed	rpm
s9	Nc	Core physical speed	rpm
s10	epr	Engine pressure ratio	—
s11	Ps30	HPC outlet static pressure	psia
s12	phi	Fuel-to-P30 ratio	pps/psi
s13	NRf	Corrected fan speed	rpm
s14	NRc	Corrected core speed	rpm
s15	BPR	Bypass ratio	—
s16	farB	Burner fuel–air ratio	—
s17	htBleed	Bleed enthalpy	—
s18	Nf_dmd	Demanded fan speed	rpm
s19	PCNfR_dmd	Demanded core speed	rpm
s20	W31	HPT coolant bleed flow	lbm/s
s21	W32	LPT coolant bleed flow	lbm/s

**Table 2:** Overall results under the same-condition setting (mean ± standard deviation over three random seeds).

**Setting**	**RMSE**	**PHM**	**PICP**	**MPIW**
FD001 → FD001 (Uncalibrated)	16.235 ± 1.297	394.9 ± 5.0	0.590 ± 0.035	22.822 ± 2.025
FD001 → FD001 (Calibrated)	16.235 ± 1.297	394.9 ± 5.0	0.800 ± 0.026	33.766 ± 0.811
FD002 → FD002 (Uncalibrated)	18.323 ± 0.411	1658.1 ± 367.2	0.722 ± 0.050	33.339 ± 4.928
FD002 → FD002 (Calibrated)	18.323 ± 0.411	1658.1 ± 367.2	0.793 ± 0.032	37.203 ± 3.583

**Table 3:** Overall results under the cross-condition setting (mean ± standard deviation over three random seeds).

Setting	RMSE	PHM	PICP	MPIW
FD001 → FD002 (Uncalibrated)	21.758 ± 1.208	2943.7 ± 782.6	0.696 ± 0.046	43.108 ± 3.900
FD001 → FD002 (Calibrated)	21.758 ± 1.208	2943.7 ± 782.6	0.806 ± 0.032	50.952 ± 0.462
FD002 → FD001 (Uncalibrated)	17.562 ± 0.062	633.3 ± 12.7	0.593 ± 0.071	25.218 ± 3.209
FD002 → FD001 (Calibrated)	17.562 ± 0.062	633.3 ± 12.7	0.803 ± 0.021	40.888 ± 2.336

**Table 4:** Baseline comparison under the same-condition setting (FD001; mean ± standard deviation over three random seeds).

FD001	RMSE	PHM	PICP	MPIW
MC Dropout-LSTM	15.456 ± 0.669	414.2 ± 3.4	0.640 ± 0.020	20.906 ± 0.913
Gaussian NLL-LSTM	19.143 ± 6.811	556.7 ± 43.2	0.807 ± 0.055	51.029 ± 19.296
TCN-QR	15.509 ± 0.568	443.3 ± 13.3	0.700 ± 0.115	28.120 ± 6.165
Deep Ensemble Gaussian-LSTM	16.007 ± 0.666	296.7 ± 6.6	0.880 ± 0.070	48.612 ± 9.386
LSTM-QR	16.235 ± 1.297	394.9 ± 5.0	0.800 ± 0.026	33.766 ± 0.811

**Table 5:** Baseline comparison under the same-condition setting (FD002; mean ± standard deviation over three random seeds).

FD002	RMSE	PHM	PICP	MPIW
MC Dropout-LSTM	17.468 ± 0.562	1740.2 ± 251.2	0.781 ± 0.046	34.238 ± 2.350
Gaussian NLL-LSTM	15.777 ± 0.673	1490.2 ± 72.1	0.747 ± 0.045	29.747 ± 2.847
TCN-QR	17.219 ± 0.378	1786.7 ± 227.2	0.708 ± 0.028	31.655 ± 2.990
Deep Ensemble Gaussian-LSTM	15.556 ± 0.064	1466.7 ± 25.2	0.762 ± 0.030	30.836 ± 2.016
LSTM-QR	18.323 ± 0.411	1658.1 ± 367.2	0.793 ± 0.032	37.203 ± 3.583

**Table 6:** Baseline comparison under the cross-condition setting (FD001 → FD002; mean ± standard deviation over three random seeds).

FD001 → FD002	RMSE	PHM	PICP	MPIW
MC Dropout-LSTM	23.932 ± 0.513	5356.7 ± 354.18	0.855 ± 0.027	54.823 ± 2.943
Gaussian NLL-LSTM	25.540 ± 1.474	1436.7 ± 202.1	0.787 ± 0.036	57.736 ± 4.985
TCN-QR	22.176 ± 0.904	2273.3 ± 511.9	0.846 ± 0.049	50.552 ± 2.525
Deep Ensemble Gaussian-LSTM	23.610 ± 0.397	1266.7 ± 225.5	0.821 ± 0.015	56.544 ± 1.124
LSTM-QR	21.758 ± 1.208	2943.7 ± 782.6	0.806 ± 0.032	50.952 ± 0.462

**Table 7:** Baseline comparison under the cross-condition setting (FD002 → FD001; mean ± standard deviation over three random seeds).

FD002 → FD001	RMSE	PHM	PICP	MPIW
MC Dropout-LSTM	39.969 ± 3.656	605.4 ± 180.4	0.823 ± 0.015	60.915 ± 5.081
Gaussian NLL-LSTM	45.240 ± 1.407	472.5 ± 35.2	0.809 ± 0.021	59.596 ± 2.017
TCN-QR	45.457 ± 2.708	585.7 ± 115.3	0.820 ± 0.017	66.487 ± 7.686
Deep Ensemble Gaussian-LSTM	44.588 ± 1.570	690.5 ± 66.4	0.807 ± 0.038	59.406 ± 2.040
LSTM-QR	17.562 ± 0.062	633.3 ± 12.7	0.803 ± 0.021	40.888 ± 2.336

**Table 8:** Ablation results of the proposed framework under the FD001 → FD002 setting. “√” means used; “–” means not used.

Setting	Low-RUL Weighting	Overestimation Penalty	Fine-Tuning	CQR	RMSE	PHM Score	PICP	MPIW
Plain QR	–	–	–	–	51.985	1,098,574.0	0.261	36.968
Weighting only	√	–	–	–	55.307	1,938,596.8	0.194	21.315
Over-penalty only	–	√	–	–	53.001	1,246,954.0	0.281	37.248
Full risk-aware	√	√	–	–	49.309	945,916.4	0.281	36.862
Plain QR + FT	–	–	√	–	20.725	3411.8	0.806	48.055
Weighting only + FT	√	–	√	–	24.374	6890.2	0.593	35.372
Over-penalty only + FT	–	√	√	–	20.841	2982.9	0.810	48.236
Full risk-aware + FT	√	√	√	–	21.050	2272.3	0.747	46.830
Plain QR + FT + CQR	–	–	√	√	20.725	3411.8	0.822	49.831
Full risk-aware + FT + CQR	√	√	√	√	21.050	2272.3	0.806	51.438

**Table 9:** Ablation results of the proposed framework under the FD002 → FD001 setting. “√” means used; “–” means not used.

Setting	Low-RUL Weighting	Overestimation Penalty	Fine-Tuning	CQR	RMSE	PHM Score	PICP	MPIW
Plain QR	–	–	–	–	48.645	61,369.6	0.260	23.683
Weighting only	√	–	–	–	48.376	62,367.8	0.280	25.494
Over-penalty only	–	√	–	–	48.481	61,657.2	0.280	27.689
Full risk-aware	√	√	–	–	47.968	57,022.9	0.250	17.297
Plain QR + FT	–	–	√	–	15.297	360.3	0.600	24.994
Weighting only + FT	√	–	√	–	14.876	338.6	0.610	25.373
Over-penalty only + FT	–	√	√	–	15.984	422.8	0.630	25.171
Full risk-aware + FT	√	√	√	–	17.491	644.2	0.530	21.969
Plain QR + FT + CQR	–	–	√	√	15.297	360.3	0.910	50.090
Full risk-aware + FT + CQR	√	√	√	√	17.491	644.2	0.810	43.554

## Data Availability

The data used in this study are publicly available from the NASA C-MAPSS dataset [https://www.kaggle.com/datasets/behrad3d/nasa-cmaps (accessed on 3 January 2026)]. The processed data and code used to support the findings of this study are available from the corresponding author upon reasonable request.

## Abbreviations

The following abbreviations are used in this manuscript:

RUL	Remaining Useful Life
LSTM	Long Short-Term Memory
QR	Quantile Regression
LSTM-QR	LSTM-based Quantile Regression
CQR	Conformalized Quantile Regression
PdM	Predictive Maintenance
